# Immune Response to Hepatitis B Vaccine in Patients with Chronic Kidney Disease

**DOI:** 10.5812/kowsar.1735143x.766

**Published:** 2011-10-01

**Authors:** Behzad Einollahi

**Affiliations:** 1 Nephrology and Urology Research Center,Baqiyatallah University of Medical Sciences, Tehran, IR Iran

**Keywords:** Hepatitis B Vaccines, Kidney Diseases

Despite the use of hepatitis B virus (HBV) vaccines and preventive mDespite the use of hepatitis B virus (HBV) vaccines and preventive measures, infection with HBV remains a major global health problem. Patients with chronic kidney disease (CKD) are at an increased risk of acquiring HBV infections from shared dialysis equipment, increased exposure to blood products, and immunodeficiency associated with CKD [1][2]. In addition, they may be more likely to develop chronic infections on exposure to HBV [1][2], and HBV infection among CKD patients is associated with high rates of morbidity and mortality [3][4]. Vaccination s important both for preventing susceptible patients from acquiring HBV and for reducing the pool of HBVinfected patients. HBV infection remains a concern in dialysis populations, because the vaccination programs have been less successful in these populations than in the general population. The causes of poor seroconversion in CKD patients include malnutrition, uremia, and immunosuppression due to renal failure [5]. In the current issue of Hepatitis Monthly, Behnam Hashemi et al. [6] compared the seroconversion rates of patients with different stages of CKD who received the HBV vaccine. The overall seroconversion rate was found to be 78%. This result was comparable with those of studies in patients with end stage renal disease (ESRD) (seroconversion rates, 60–90.5%) [7]. The HBV vaccination is less effective in patients with ESRD than in those without CKD [7]. The seroconversion rate among individuals without CKD was over 90% whereas that among patients with ESRD was only 50–60% [8]. In addition, compared to patients without CKD, ESRD patients frequently showed lower seroconversion rates, lower peak antibody titers, and a more rapid decline of antibody levels [9]. Behnam Hashemi et al. showed that there was significant correlation between hepatitis B surface (HBs) antibody titer and the glomerular filtration rate (GFR) (P = 0.001). However, there was no evident correlation between the seroconversion rate and severity of renal dysfunction. They showed that HBV vaccination in the early stages of CKD did not result in significantly higher seroconversion rates than those achieved on HBV vaccination in the late stages of CKD. Another stu   showed that there was no association between the response rate and degree of renal dysfunction in individuals with mild to moderate CKD [10]. However, several studies showed that ESRD patients who were vaccinated before they required dialysis showed higher seroconversion rates and antibody titers than those who were undergoing dialysis when they received vaccination, suggesting a correlation betweenimmune response and degree of renal dysfunction [5][7][11]. It is very important that HBV vaccination be carried out in the early stage of CKD, since seroconversion is correlated with the amount of GFR [5]. DaRoza et al. in a large cohort study showed that patients with higher GFR levels showed a better immune response to the HBV vaccine [5]. These diverse findings may be partly attributable to different inclusion criteria; for example, the ESRD patients included in the study by DaRoza et al. [5] had a poorer GFR than that of the patients in the study by McNulty et al [10]. Nonetheless, the strategies recommended for improving seroconversion rates among ESRD patients include vaccination in the early stages of CKD [12]. Furthermore, elderly patients with ESRD seemed to have a lower rate of seroconversion than that in the younger patients [10][11][12][13]. Behnam Hashemi et al., showed a significant negative correlation between old age (> 60 years) with seroconversion rate. Advanced age, male gender, diabetes CKD patients, and previous blood transfusions are associated with poor seroconversion rates [7][8][9][10][11][12][13][14]. Advanced age   gatively affected the seroconversion rate regardless of the patient’s status [5][7][15]. In conclusion, HBV vaccination is necessary in CKD patients, because they are at a high-risk of developing HBV infections. Despite these controversial findings, many clinicians recommend that patients with CKD receive vaccination as soon as chronic progressive renal failure is diagnosed, and if possible, before dialysis is required.

## References

[1] Chacko EC, Surrun SK, Mubarack Sani TP, Pappachan JM (2010). Chronic viral hepatitis and chronic kidney disease. Postgrad Med J.

[2] Peters MG (2009). Special populations with hepatitis B virus infection. Hepatology.

[3] Molino C, Fabbian F, Cozzolino M, Longhini C (2008). Peters MG.The management of viral hepatitis in CKD patients: an unresolved problem. Int J Artif Organs.

[4] Fabrizi F, Messa P, Martin P (2008). Hepatitis B virus infection and the dialysis patient. Semin Dial.

[5] DaRoza G, Loewen A, Djurdjev O, Love J, Kempston C, Burnett S, Kiaii M, Taylor PA, Levin A (2003). Stage of chronic kidney disease predicts seroconversion after hepatitis B immunization: earlier is better. Am J Kidney Dis.

[6] Hashemi B, Mahdavi Mazdeh M, Abbasi M, Hosseini Moghaddam SM, Hatmi Zinat N, Ahmadi F (2011). Efficacy of HBV Vaccination in Various Stages of Chronic Kidney Disease: Is Earlier Better?. Hepat Mon.

[7] Janus N, Vacher LV, Karie S, Ledneva E, Deray G (2008). Vaccination and chronic kidney disease. Nephrol Dial Transplant.

[8] Buti M, Viladomiu L, Jardi R, Olmos A, Rodriguez JA, Bartolome J, Esteban R, Guardia J (1992). Long-term immunogenicity and efficacy of hepatitis B vaccine in hemodialysis patients. Am J Nephrol.

[9] Fabrizi F, Martin P (2001). Hepatitis B vaccine and dialysis: current issues. Int J Artif Organs.

[10] McNulty CA, Bowen JK, Williams AJ (2005). Hepatitis B vaccination in predialysis chronic renal failure patients a comparison of two vaccination schedules. Vaccine.

[11] Kausz A, Pahari D (2004). The value of vaccination in chronic kidney disease. Semin Dial.

[12] Agarwal S (2010). Hepatitis B Infection during Renal Replacement Therapy. Hepat Mon.

[13] Kara IH, Yilmaz ME, Suner A, Kadiroglu AK, Isikoglu B (2004). The evaluation of immune responses that occur after HBV infection and HBV vaccination in hemodialysis patients. Vaccine.

[14] Alavian SM, Tabatabaei SV (2010). The effect of diabetes mellitus on immunological response to hepatitis B virus vaccine in individuals with chronic kidney disease: A meta-analysis of current literature. Vaccine.

[15] Fabrizi F, Martin P, Dixit V, Bunnapradist S, Dulai G (2004). Meta-analysis: the effect of age on immunological response to hepatitis B vaccine in end-stage renal disease. Aliment Pharmacol Ther.

